# Molecular Characteristics of Extraintestinal Pathogenic *E. coli* (ExPEC), Uropathogenic *E. coli* (UPEC), and Multidrug Resistant *E. coli* Isolated from Healthy Dogs in Spain. Whole Genome Sequencing of Canine ST372 Isolates and Comparison with Human Isolates Causing Extraintestinal Infections

**DOI:** 10.3390/microorganisms8111712

**Published:** 2020-10-31

**Authors:** Saskia-Camille Flament-Simon, María de Toro, Vanesa García, Jesús E. Blanco, Miguel Blanco, María Pilar Alonso, Ana Goicoa, Juan Díaz-González, Marie-Hélène Nicolas-Chanoine, Jorge Blanco

**Affiliations:** 1 Laboratorio de Referencia de E. coli (LREC), Departamento de Microbioloxía e Parasitoloxía, Facultade de Veterinaria, Universidade de Santiago de Compostela (USC), 27002 Lugo, Spain; saskia.flament@usc.es (S.-C.F.-S.); vanesag.menendez@usc.es (V.G.); jesuseulogio.blanco@usc.es (J.E.B.); miguel.blanco@usc.es (M.B.); juan.diaz.glez@gmail.com (J.D.-G.); 2 Instituto de Investigación Sanitaria de Santiago de Compostela (IDIS), 15706 Santiago de Compostela, Spain; pilar.alonso.garcia@sergas.es; 3 Plataforma de Genómica y Bioinformática, Centro de Investigación Biomédica de La Rioja (CIBIR), 26006 Logroño, Spain; mthernando@riojasalud.es; 4 Unidade de Microbioloxía, Hospital Universitario Lucus Augusti (HULA), 27003 Lugo, Spain; 5 Servicio de Medicina Interna, Hospital Veterinario Universitario Rof Codina, USC, 27002 Lugo, Spain; anagoicoavaldevira@gmail.com; 6 Departamento de Anatomía, Producción Animal e Ciencias Clínicas Veterinarias, USC, 27002 Lugo, Spain; 7 Université de Paris, IAME, UMR1137, INSERM, F-75018 Paris, France; marie-helene.nicolas-chanoine@inserm.fr

**Keywords:** *Escherichia coli*, dogs, virulence genes, antibiotic resistance, WGS, ST372, clonal structure

## Abstract

Under a one health perspective and the worldwide antimicrobial resistance concern, we investigated extraintestinal pathogenic *Escherichia coli* (ExPEC), uropathogenic *E. coli* (UPEC), and multidrug resistant (MDR) *E. coli* from 197 isolates recovered from healthy dogs in Spain between 2013 and 2017. A total of 91 (46.2%) isolates were molecularly classified as ExPEC and/or UPEC, including 50 clones, among which (i) four clones were dominant (B2-CH14-180-ST127, B2-CH52-14-ST141, B2-CH103-9-ST372 and F-CH4-58-ST648) and (ii) 15 had been identified among isolates causing extraintestinal infections in Spanish and French humans in 2015 and 2016. A total of 28 (14.2%) isolates were classified as MDR, associated with B1, D, and E phylogroups, and included 24 clones, of which eight had also been identified among the human clinical isolates. We selected 23 ST372 strains, 21 from healthy dogs, and two from human clinical isolates for whole genome sequencing and built an SNP-tree with these 23 genomes and 174 genomes (128 from canine strains and 46 from human strains) obtained from public databases. These 197 genomes were segregated into six clusters. Cluster 1 comprised 74.6% of the strain genomes, mostly composed of canine strain genomes (*p* < 0.00001). Clusters 4 and 6 also included canine strain genomes, while clusters 2, 3, and 5 were significantly associated with human strain genomes. Finding several common clones and clone-related serotypes in dogs and humans suggests a potentially bidirectional clone transfer that argues for the one health perspective.

## 1. Introduction

*Escherichia coli* is a common commensal of the gastrointestinal tract. However, *E. coli* is also the main bacterial pathogen responsible for extraintestinal infections in humans and dogs, including urinary tract infections (UTIs) [1,2,3,4,5]. Most UTIs are thought to result from ascending infections. The two theories for the origin of uropathogenic isolates are the “prevalence” and the “special pathogenicity”. The prevalence hypothesis postulates that most UTIs are opportunistic infections caused by bacteria that predominate in the faecal microbiota, whereas the special pathogenicity hypothesis suggests that most UTI are caused by pathogenic strains that possess appropriate virulence factor (VF)-encoding genes [5,6]. More than 50 *E. coli* genes associated with extraintestinal infections have been identified, encoding adhesins, toxins, siderophores, capsular antigens, and invasines [7,8]. Isolates are designed presumptively as extraintestinal pathogenic *E. coli* (ExPEC) if they contained ≥2 of 5 of the following VF-encoding genes: *papAH* and/or *papC*, *sfa/focDE*, *afa/draBC*, *kpsM II*, and *iutA* [7], and as uropathogenic *E. coli* (UPEC) if they are positive for ≥3 of the four following VF-encoding genes: *chuA*, *fyuA*, *vat*, and *yfcV* [8].

The majority of ExPEC and UPEC isolates belong to B2 phylogenetic group. Although there is a notable diversity of phylogenetic groups among *E. coli* isolates causing human and animal extraintestinal infections, some epidemiological studies indicate that certain O:H serotypes, sequence types (STs) and clonotypes are more predominant and especially successful [3,9,10,11,12,13,14,15]. Three recent studies showed the dominance of some STs in dogs in Australia, the United States, and France, such as ST372, assessed to be specifically associated with dogs, and ST12, ST73, ST127, and ST141, assessed to be specifically associated with humans [10,11,14]. On the other hand, within-household sharing of ExPEC ST73 and ST95 strains, those with same serotypes and VF-encoding genes have been documented in the United States among humans and dogs [16]. Furthermore, in Australia and the United States, human and canine *E. coli* ST127, ST131, and ST1193 that exhibited identical virulence genotypes and highly similar PFGE profiles have been identified [17,18,19]. These findings suggest that some *E. coli* infections may sometimes be a zoonosis in either direction (human to pet or pet to human).

The antimicrobial resistance of human ExPEC and UPEC isolates has increased dramatically due to the emergence of the pandemic clone ST131 and more especially to subclade C2 (also known as subclone *H*30Rx) [20,21,22,23,24,25,26]. This subclone has also been occasionally isolated from dogs in several countries [27,28,29,30,31]. The emergence of multidrug resistance (MDR) among *E. coli* causing infections in dogs is of great concern and increases the risk of treatment failure [32,33,34,35,36,37,38,39,40,41,42]. Additionally, exposure to dogs and/or dog faeces has been identified as a risk factor for the development of drug-resistant *E. coli* UTI in women [43].

As relatively little is known on the clonal structure of canine ExPEC, UPEC and MDR isolates, the present study was carried out (i) to establish which clones (defined by the association of phylogroup, clonotype and ST) dominate in dogs and (ii) to compare these clones with those causing extraintestinal infections in humans. To our knowledge, this is the first study that uses whole genome sequencing (WGS) to define the genetic relatedness between the ST372 *E. coli* lineage, which we found dominant among the Spanish canine faecal *E. coli* populations, and human *E. coli* ST372 that cause extra-intestinal infections.

## 2. Materials and Methods

### 2.1. E. coli Isolates

A total of 197 non-duplicate *E. coli* isolated from faecal samples of 104 healthy dogs collected in Spain between 2013 and 2017 were characterized.

### 2.2. Phylogenetic Grouping

Assignment to the main phylogroups (A, B1, B2, C, D, E, and F) was based on the protocol of Clermont et al. [44].

### 2.3. Serotyping

The determination of O and H antigens was carried out using the method previously described by Guinée et al. [45] with all available O (O1 to O181) and H (H1 to H56) antisera. Isolates that did not react with any antisera were classified as O non-typeable (ONT) or H non typeable (HNT) and those non motile were denoted as HNM.

### 2.4. Multilocus Sequence Typing (MLST)

The sequence types (STs) were established following the MLST scheme of Achtman by gene amplification and sequencing of the seven housekeeping genes (*adk*, *fumC*, *gyrB*, *icd*, *mdh*, *purA*, and *recA*) according to the protocol and primers specified at the *E. coli* MLST web site (http://mlst.warwick.ac.uk/mlst/dbs/Ecoli) [46].

### 2.5. CH Typing

Clonotype identification was determined by *fumC* and *fimH* (CH) sequencing [47,48].

### 2.6. Virulence Genotyping

VF-encoding genes of *E. coli* causing extraintestinal infections were screened by PCR [4,49]. The virulence gene score was the number of extraintestinal virulence-associated genes detected. The isolates were designed presumptively as extraintestinal pathogenic *E. coli* (ExPEC) if positive for ≥2 of 5 markers, including *papAH* and/or *papC*, *sfa/focDE*, *afa/draBC*, *kpsM II*, and *iutA* [7], and as uropathogenic *E. coli* (UPEC) if positive for ≥3 of 4 markers, including *chuA*, *fyuA*, *vat*, and *yfcV* [8].

### 2.7. Antimicrobial Susceptibility and ESBL and pAmpC Typing

Antimicrobial susceptibility was determined by the minimal inhibitory concentrations (MICs) and/or the disc diffusion method. Resistance was interpreted based on the recommended breakpoints of the CLSI [50]. Fifteen classes of antimicrobial agents were analyzed: penicillins (ampicillin), penicillins and β-lactamase inhibitors (amoxicillin-clavulanic acid), 1st and 2nd generation of non-extended spectrum cephalosporins (cefazolin and cefuroxime), extended-spectrum cephalosporins (cefotaxime, ceftazidime and cefepime), cephamycins (cefoxitin), monobactams (aztreonam), carbapenems (imipenem), aminoglycosides (gentamicin, tobramycin, amikacin), tetracyclines (doxycycline), phenicols (chloramphenicol), nitrofurans (nitrofurantoin), quinolones (nalidixic acid and ciprofloxacin), folate pathway inhibitors (trimethoprim-sulphamethoxazole), phosphonic acids (fosfomycin), and polymyxins (colistin). *E. coli* MDR was defined as resistance to one or more agents in three or more classes of tested drugs [51]. Genetic identification of ESBL and pAmpC types was carried out by PCR followed by amplicon sequencing [52,53,54].

### 2.8. Whole Genome Sequencing (WGS)

The WGS of 23 ST372 isolates from our LREC collection was performed under the protocol of the Genomics and Bioinformatics Core Facility (Centre for Biomedical Research of La Rioja) as it was described previously [55]. The assembly information of draft genomes, database sources and input parameters can be found in Table S1 (NCBI Bioproject accession PRJNA627579).

PLACNET webserver [56,57] was used for the genome reconstruction after which Prokka [58] was used to annotate the assembled genetic elements. Primary in silico analyses were carried out using the Center for Genomic Epidemiology (CGE) (http://www.genomicepidemiology.org/) services with home-made databases, the CGE databases, and other complementary databases to explore the resistance and virulence factors. Plasmid typing was complemented by subtyping relaxases with the method defined by Alvarado et al. [59] and integrative conjugative elements (ICEs) typing was complemented by in silico analyzing the ICE-harbouring contigs with ICEberg (ICEfinder and VRprofile) (information provided in Table S1). Additionally, the ICE-harbouring contigs were analyzed with Easyfig, a comparative genomic tool that allows for visualizing homologies and similarities between contigs using BLAST [60].

Besides, we performed a single nucleotide position (SNP) tree analysis of the 23 ST372 genomes sequenced in this study plus 174 ST372 full-genome references retrieved from NCBI bioproject and EnteroBase. The SNP-tree was done using the CSI Phylogeny 1.4 server from the CGE with J22 strain as reference (ID: GCA_009497315). After analyzing the SNP matrix, we took all the ST372 genomes from human strains plus some representative genomes from canine strains to make a tree visualization using EnteroBase [61]. The accession number of all the genomes included in this study can be found in Table S2.

### 2.9. Statistical Analysis

All the *p* values were calculated using Fisher’s exact test, except for the comparison of the means that was performed using the one-way ANOVA test. *p* values < 0.05 were considered statistically significant.

## 3. Results

### 3.1. Phylogenetic Groups of the 197 Canine Isolates

The most common phylogenetic group displayed by the 197 canine faecal *E. coli* isolates was B2 (42.6%), followed by A (16.2%), B1 (13.2%), F (9.1%), E (7.1%), C (5.1%), and D (3.0%) (Table S3).

### 3.2. Virulence Factor (VF)-Encoding Genes in the 197 Canine Isolates

Of the 28 VF-encoding genes analyzed, eight (*fimH*, *yfcV*, *vat*, *iroN*, *fyuA*, *chuA*, *malX*, and *usp*) were detected in more than 40% of the 197 canine isolates and nine (*papAH*, *papC*, *sfa/focDE*, *cnf1*, *hlyA*, *kpsM II*, *kpsM II-K5*, *traT*, *ibeA*) in at least 20%. In contrast, six VF-encoding genes (*afa/draBC*, *sat*, *cdtB*, *neuC-K1*, *kpsM II-K2*, *kpsM III*) were found in less than 10% of these isolates (Table 1).

A higher mean of VF-encoding gene score was observed in the 84 canine isolates belonging to the dominant B2-phylogenetic group (mean of 12.79) (*p* < 0.05) compared with the isolates belonging to phylogroups A (2.31), B1 (2.96), C (6.60), D (5.67), E (4.14), and F (7.94) (Table 1).

Of the 197 canine isolates, 74 (37.6%) were presumptively classified as ExPEC and 82 (41.6%) as UPEC (Table 1) resulting in 91 ExPEC and/or UPEC isolates. The majority (85.7%; 78 of 91) of ExPEC and/or UPEC isolates belonged to phylogenetic group B2. In contrast, only 5.7% (6 of 106) of non-ExPEC and non-UPEC isolates were assigned to this phylogenetic group (*p* < 0.00001). The A, B1, C, and E phylogenetic groups were significantly associated with non-ExPEC and non-UPEC isolates (Table S4).

### 3.3. Antimicrobial Resistance in the 197 Canine Isolates

In total, 28 (14.2%) of the 197 analyzed canine faecal *E. coli* isolates were classified as MDR. Multidrug resistance was significantly associated with isolates belonging to B1, D, and E phylogenetic groups (Table S5). Furthermore, only eight (28.6%) of MDR isolates showed the ExPEC and/or the UPEC status (Table S6).

In total, 10 of the 28 MDR isolates produced an ESBL enzyme: CTX-M-1 (four isolates), CTX-M-14 (four isolates), CTX-M-55 (one isolate) and SHV12 (one isolate). Besides, 10 other isolates produced a plasmid-mediated AmpC β-lactamase of CMY-2 type.

### 3.4. Sequence Types, Clones and Serotypes Displayed by the 91 Canine ExPEC and/or UPEC Isolates and 28 MDR Isolates

Sequences types (STs), clones (defined by the association of phylogroup, clonotype and ST) and O:H serotypes were established only for the 91 canine isolates classified as ExPEC and/or UPEC and the 28 canine MDR isolates.

A total of 34 STs were identified in the canine ExPEC and/or UPEC isolates and 22 in the MDR isolates. Among these STs, 18 were previously undescribed (Table S7). Each of these 18 new STs were displayed by one isolate. Seven dominant STs (ST12, ST38, ST73, ST127, ST141, ST372, and ST648) were observed among the 91 canine ExPEC and/or UPEC and the 28 canine MDR isolates. There was a strong correlation between VF-encoding gene profiles and the dominant STs (Table 2).

A total of 50 clones were identified among the 91 canine isolates classified as ExPEC and/or UPEC, with 11 of them including at least two isolates and only four, at least four isolates i.e., B2-CH14-180-ST127 (four isolates), B2-CH52-14-ST141 (four isolates), B2-CH103-9-ST372 (25 isolates), and F-CH4-58-ST648 (five isolates) (Table 3). In recent studies conducted by our research group [3,21,62], we had identified, as indicated in Table 3, 15 of the 50 canine ExPEC/UPEC clones comprising 49 isolates among the 261 human ExPEC and/or UPEC isolates included in a collection of 394 *E. coli* isolates causing extraintestinal infections. However, only 31 of the 49 human ExPEC and/or UPEC isolates presented the same clone-related O:H serotypes as the canine isolates (Table 3). Among these 31 human isolates, 28 belonged to B2 phylogroup clones and three to F phylogroup clones identified among canine isolates. These B2 clones were distributed into five ST lineages, including four lineages currently dominant in humans (ST73, ST127, ST141, and ST1193,) and the lineage currently established as the dominant lineage in dogs, namely lineage ST372. In dogs, we found three clones in the lineage ST73 with the same serotype (O6:H1). The eight human isolates sharing this lineage with dogs were distributed into the same three clones and displayed serotype O6:H1. In dogs, we found two clones in the lineage ST127 displaying two serotypes with one (O6:HNM) of them present in the two clones. The four human isolates sharing this lineage with dogs were distributed into the same two clones but displayed the common serotype (O6:HNM). In dogs, we found two clones in the lineage ST141 with the same serotype (O2:H6). The 11 human isolates that shared this lineage with dogs were distributed into the same two clones and displayed the same serotype as human isolates. In dogs, we found one clone in lineage ST1193 with one serotype (O75:HNM). Three human isolates shared this clone and serotype with dogs. Concerning the lineage ST372, we found five clones in dogs with one of them including isolates displaying six serotypes. The two human isolates sharing the lineage ST372 with dogs belonged to this multiple-serotype clone and both displayed one of the six serotypes (O83:H31). Concerning the three F group human isolates, they belonged to one of the three F group clones (F-CH32-41-ST59) identified in dogs and showed the same serotype (O1:H7).

Among the 28 canine MDR isolates, we observed 24 different clones, of which nine had also been identified among the above cited 394 isolates causing infections in humans (Table 4) [3,21,62].

### 3.5. Whole Genome Sequencing (WGS) and Molecular Characterisation of ST372 Isolates

For WGS, we selected 23 of the above studied ST372 isolates. They comprised 21 of the 29 Spanish canine faecal ST372 strains that were isolated in 2013 (n = 9) and 2017 (n = 12) and two previously published human ST372 strains isolated in 2016 [3,61]: strains LREC_341 isolated in Spain from an abscess and LREC_342 isolated in France from a bone infection. Both human strains showed serotype O18:H31 and clonotype CH103-9, whereas the 21 canine strains showed six different serotypes (O4:H31 (seven isolates), O83:H31 (four isolates), O25:H31 (four isolates), O15:H31 (three isolates), O21:H31 (two isolates) and O117:H28 (one isolate)) and four clonotypes (CH103-9 (18 isolates), CH103-10, CH103-17, and CH103-240).

The main objectives were to get more insights into the *E. coli* ST372 lineage that appears as one of the most prevalent *E coli* lineages among the canine faeces *E. coli* populations and to elucidate if there is any relation between canine and human ST372 strains.

To infer the phylogeny, we performed an SNP-tree with 197 genomes of ST372 strains (23 from this study (labelled LREC strains) and 174 obtained from public databases) corresponding to 151 genomes from canine strains and 46 genomes from human strains. A total of 70% of these genomes corresponded to strains collected between 2017 and 2019 while the remaining 30% corresponded to strains isolated between 1995 and 2016. Regarding geographical distribution, 46 genomes (23.4%) were from strains collected in Europe and 143 (72.6%) from strains collected in North America.

**Figure 1:**
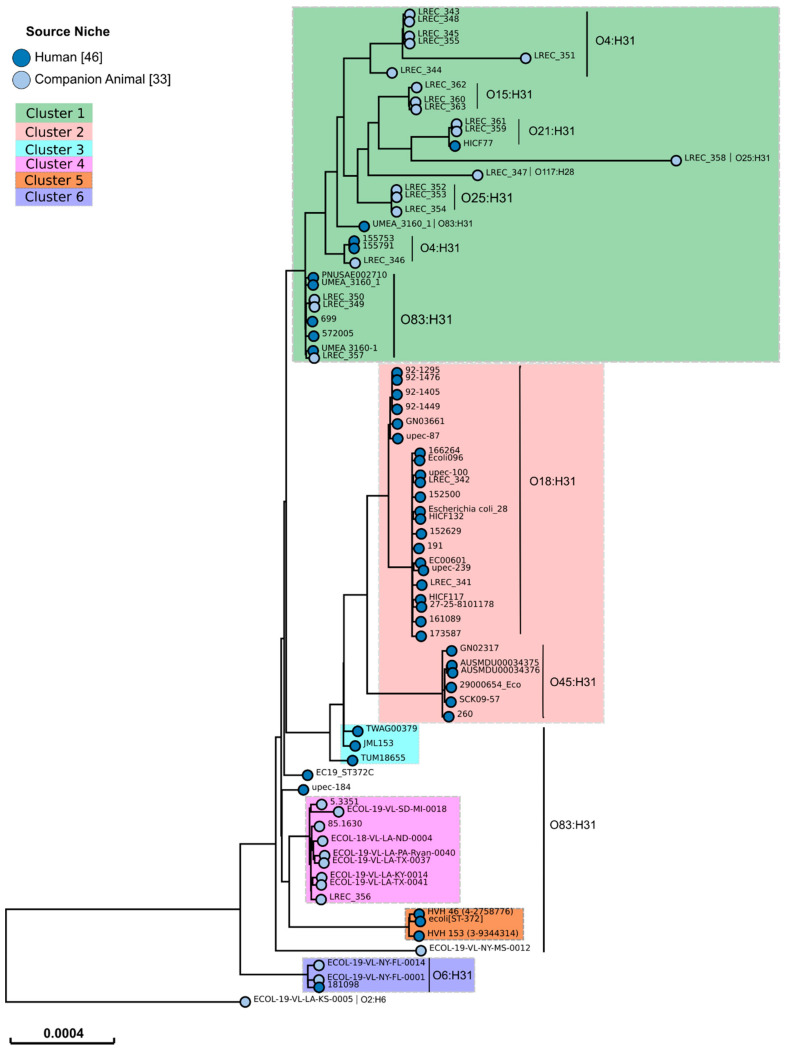
SNP-tree of 79 representative ST372 *E. coli* genomes from 46 human strains and 33 canine strains. Tree visualization by EnteroBase [61]. The 33 genomes from canine strains are representatives of the different clusters identified in the SNP matrix of a previous SNP-tree performed with the 197 genomes analyzed in this study. The identified serotypes are listed beside the vertical line.

The SNP analysis of the *E. coli* ST372 lineage revealed a wide and heterogeneous population, allowing us to describe six clusters. Figure 1 only includes 97 representative genomes (including the 23 LREC genomes sequenced in this study and the 46 genomes from human strains) of the 197 analyzed so that it is possible to visualize all the information.

The criterion established to define a cluster was that it should include genomes with less than 200 SNPs distance between them. An exception to this rule was the inclusion of the genome ECOL-19-VL-SD-MI-0018, with a maximum of 391 SNP distance, in cluster 4. Five genomes did not reach this criterion, having more than 400 SNP distance between them and could form five other clusters. However, we have included those genomes in only one category (undefined) to simplify the following analysis.

According to the phylogenetic tree built from the genome of the 197 strains, cluster 1 comprised 147 (74.6%) of the 197 analyzed genomes. This cluster was mostly composed of genomes of canine strains (138 genomes; 93.9%). Genomes of canine strains were also included in clusters 4 (nine genomes) and 6 (two genomes) while only human strain genomes were included in clusters 2 (28 genomes), 3 (three genomes) and 5 (three genomes) (Table 5). Thus, cluster 1 comprised significantly more canine strain genomes (*p* < 0.00001) while clusters 2 (*p* < 0.00001), 3 (*p* = 0.01209), and 5 (*p* = 0.01209) comprised significantly more human strain genomes. A total of 20 of the 21 genomes of the Spanish canine strains belonged to cluster 1, whereas, the genome of the remaining Spanish canine strain (LREC_356) belonged to cluster 4. The genomes of the Spanish and French human strains (LREC_341 and LREC_342) belonged to cluster 2.

Both clusters 1 and 2 were the most frequent clusters observed among the studied *E. coli* ST372 strains (canine and human) isolated in Europe and North America. However, cluster 1 was significantly associated with North America strains (*p* = 0.02476), while cluster 2 was especially associated with Europe strains (*p* = 0.01233) (Table 6).

To compare the virulence profile of the 197 canine and human ST372 strains, we in silico investigated the presence of 32 VF-encoding genes in the 197 strains and defined their ExPEC and UPEC status. We also investigated the distribution of those VF-encoding genes according to the classification of the strains into the six defined clusters. Table 7 summarizes the results obtained from the mentioned analysis. Microbiological, geographical, and genomic data of each of the 197 studied strains are available in Table S2.

The canine ST372 strains showed a higher VF-encoding gene score (mean 16.79) compared with the human ST372 strains (mean 13.76). However, three human stains belonging to cluster 5 were those with the highest number of VF-encoding genes (mean 21.67). Eight VF-encoding genes (*papAH*, *papC*, *papEF*, *focCD*, *focG*, *cnf1*, *hlyA*, and *iroN*) were significantly associated with canine ST372 isolates, whereas five (*hlyF*, *iutA*, *kpsM II*, *kpsM II-K5*, and *iss1*) were significantly associated with human ST372 isolates. Interestingly, the ExPEC status was found more frequently among canine ST372 strains (74.8%) than human strains (21.7%) (*p* < 0.00001) (Table 7).

The more prevalent serotype was O83:H31, which represents 36.0% of the 197 ST372 strains, followed by O4:H31 (17.8%), O15:H31 (15.2%), O18:H31 (10.2%), O45:H31 (3.0%), O117:H28 (3.0%), O21:H14 (2.5%), O21:H31 (2.5%), O75:H31 (2.5%), O-unknown:H31 (2.5%), O25:H31 (2.0%), O2:H6 (0.5%), and O-unknown:H28 (0.5%). The serotypes O4:H31 (*p* = 0.02631) and O15:H31 (*p* = 0.00062) were significantly associated with canine ST372 strains, whereas the serotypes O18:H31 (*p* < 0.00001) and O45:H31 (*p* = 0.00012) were significantly more frequent among human ST372 strains. The 65 canine strains of serotypes O4:H31 and O15:H31 belonged to cluster 1 and the 26 human strains of serotypes O18:H31 and O45:H31 belonged to cluster 2 (Table 8). In contrast, the dominant serotype O83:H31 was frequently identified among canine (38.4%) and human (28.3%) strains, and, although the majority of the strains with this serotype belonged to cluster 1, O83:H31 strains were also found in clusters 3, 4, and 5.

The three most prevalent serotypes in Europe were O4:H31 (23.9%), O83:H31 (21.7%) and O18:H31 (19.6%), while in North America, they were O83:H31 (39.9%), O15:H31 (18.2%) and O4:H31 (16.8%). The serotypes O18:H31 (*p* = 0.02203) and O25:H31 (*p* = 0.00317) were more frequently observed in Europe, whereas the serotype O83:H31 (*p* = 0.03291) was more prevalent in North America (Table 9).

The 23 LRCE genomes sequenced in this study were investigated in greater depth. These genomes were reconstructed to analyze the chromosome and plasmidome separately. The size of the chromosomes had an average of 5,043,308 pb and were encompassed in 55 to 178 contigs. We found an integrative conjugative element (ICE) with relaxase type MOB_Q_ in all the genomes except for LREC_347 genome. These ICEs belong to the ICEKp1 family, a yersiniabactin synthesis-associated ICE type (similar to ICEEcoUMN026-1). The contigs that harboured the ICE region were revised, allowing us to detect the presence of a pathogenic island (PAI) and some VF-encoding genes. The ICE contig retrieved from LREC_356 genome was the longest (2,540,863 pb) and showed a high percentage of homology with the contigs of the other genomes harbouring the ICE region (Figure S1). Interestingly, the contig from LREC_356 harboured the *ompT*, *iss*, *vat*, *fyvA*, and *yfcV* virulence- encoding genes, the last three mentioned genes being those used (in addition to *chuA*) to define UPEC status. We also identified the secretion system effector homolog type T6SS and the PAI_AET37190. In second place, in terms of ICE contig length, was LREC_357 (1,677,509 pb) genome that harboured the *iroN*, *iss*, *fyvA*, and *yfcV* virulence-encoding genes. The ICE contigs had not the same length [varying from 2,540,863 pb to 307,789 pb (short read sequencing limitations)] and all the genes mentioned as found in the ICE contig from LREC_356 were not retrieved in the other 20 genomes harbouring ICE contigs: three harboured the *fyvA* and *yfcV* genes (LREC_359, LREC_361, and LREC_344) and the remaining 17 genomes harboured the *fyvA* gene. We concluded that the presence of this type of ICE was a common feature in the ST372 genomes from the 22 of 23 studied Spanish strains and may be involved in the acquisition of their UPEC status.

We also described 11 plasmids (four conjugative plasmids, six mobilizable plasmids and one plasmid with no relaxase suggesting that it is not mobilizable) which belonged to the following relaxase families (MOB) and incompatibility groups (Inc.): MOB_P3_/IncX1 (n = 3); MOB_P1_/nd (n = 2); MOB_F12_/IncFII-pCD1 (n = 2); MOB_F12_/IncFII-IncFIB (n = 1); MOB_H11_/IncHI2 (n = 1); MOB_Qu_/ColRNAI (n = 1); nd/p0111 (n = 1). To predict plasmid transferability, we investigated the presence of mating pair formation (Mpf) system proteins. These proteins were present in all the previously described MOB_F12_ and MOB_H11_ conjugative plasmids. Furthermore, in silico analysis showed that these plasmids did not carry resistance or virulence encoding genes except for the *cba* and *cma* genes that were found in plasmid pLREC354_1 and a *bla*_TEM_ gene found in pLREC346_1. Table 10 summarizes the MGE content of the 23 ST372 genomes.

We in silico investigated the presence of 189 VF-encoding genes, 87 antibiotic-resistance encoding genes (ARGs), and 18 types of point mutations (Table S8). Through this analysis, the 23 ST372 strains were shown with an UPEC status and harbouring a wide variety of VF-encoding genes, reaching an average number of 80. In contrast, these 23 ST372 strains were shown as carrying very few ARGs. However, genes encoding drug efflux were detected but only in the two human strain genomes (LREC_341 and LREC_342) that also harboured antibiotic-resistance encoding genes: *bla_TEM-1A_*, *sul1*, *aadA1*, *dfrA1*, and *mdf(A)*. These results were in agreement with those previously obtained by conventional methods.

## 4. Discussion

To get more insights into the population structure of canine *E coli*, we investigated those harboured in the intestinal tract of 104 healthy Spanish dogs by using different approaches, knowing that the gut is the reservoir of the great majority of *E. coli* causing extraintestinal infections. The phylogenetic group, VF-encoding gene and antibiotic susceptibility analyses, showed that among the 197 canine faecal isolates obtained from the 104 dogs, 84 (42.6%) belonged to B2 phylogroup, 91 (46.2%), mostly B2 group isolates, were classified as ExPEC and/or UPEC, and 28 (14.2%), mostly non-B2 group isolates, as MDR. This strongly suggests that the intestinal tract of healthy dogs might be an important reservoir of ExPEC and/or UPEC isolates, and in a lesser extent, of MDR *E. coli* isolates. However, some studies that focused on antibiotic-resistant canine isolates suggested that dogs might also be an important reservoir for antibiotic-resistant strains [63,64,65,66,67,68,69,70,71,72,73,74,75,76,77,78,79,80,81], notably for those producing ESBLs or CMY-2 [64,66,67,70,72]. Although there was a low prevalence of MDR isolates among the 197 studied isolates, we found, as previously described that they produced ESBLs or CMY-2.

MLST assigned the 91 Spanish canine faecal isolates with an ExPEC and/or UPEC status to 34 STs. among which six were displayed by 67% of the 91 isolates: ST372 (31.9%), ST12 (9.9%), ST127 (8.8%), ST648 (6.6%), ST141 (5.5%), and ST73 (4.4%). Few studies have been carried out so far to characterize the ST structure of canine ExPEC and/or UPEC isolates. In the USA, LeCuyer et al. [11] analyzed 295 *E. coli* isolates from canine UTI. They found that ST372, which is uncommon among the human *E. coli* pathogens [3,12,62,82,83], was the predominant ST in canine UTI isolates (21.7%), and this was well ahead of the five other most frequent STs: ST12 (6.4%), ST73 (6.4%), ST127 (4.1%), ST131 (4.1%), and ST297 (3.7%). A total of 170 (57.4%) of these isolates met the criterion to be classified as ExPEC, and, except for ST297, the most prevalent STs were associated with ExPEC status. In France, Valat et al. [14] analyzed 618 canine *E. coli* isolates collected from diagnostic laboratories, including 403 (65.2%) from UTIs. B2 phylogroup was over-represented (79.6%) and positively associated with the presence of numerous VFs, including those defining the ExPEC status. MLST of a randomly chosen subset of 89 isolates belonging to B2 phylogroup revealed five dominant STs: ST372 (17.9%), ST73 (17.9%), ST12 (10.1%), ST141 (7.9%), and ST961 (5.6%). In Australia, Kidsley et al. [10] focused their study on the canine fluroquinolone-susceptible *E. coli* clinical isolates (n = 449) that were identified during a nation-wide survey of antibiotic resistance in Australian animals between January 2013 and January 2014. They found that these isolates mostly (n = 317; 71%) belonged to B2 phylogroup. By using the RAPD typing system, they found a distribution of the 317 B2 group isolates into 35 main clusters. To pursue their molecular investigation, they sequenced and analyzed the whole genome of 77 representatives of the B2 group fluoroquinolone-susceptible isolates. Thus, they found that the 77 sequenced isolates were assigned to 24 STs, among which four were dominant: ST372 (31%), ST73 (17%), ST12 (7%), and ST80 (7%). In sum, the present study and those previously published show that three STs (ST372, ST12, and ST73) are the dominant ST in healthy and infected dogs irrespective of the countries (the USA, France, Australia and Spain) and strain sources (clinical samples and faeces). Such a finding might argue for the “prevalence” theory with regard to UTI pathogenesis (most UTIs are opportunistic infections caused by bacteria that predominate in the faecal microbiota) in dogs. Nevertheless, the fact that the canine isolates belonging to the most dominant ST, either present in all studied countries (ST372, ST12, and ST73) or present in some studied countries (ST127 in the USA and Spain, and ST141 in France and Spain) were shown to harbour numerous VF-encoding genes might also argue for the “special pathogenicity” theory in dogs.

Concerning the ST structure of canine MDR isolates, there seems to exist a more important difference between the countries than for the non-MDR isolates. MLST assigned the 28 Spanish canine MDR isolates to a great diversity of STs comprising 15 established STs (ST10, ST12, ST38, ST57, ST58, ST88, ST93, ST155, ST457, ST648, ST695, ST1011, ST1140, ST3774, and ST8953) and seven new STs. The first 10 STs here listed have been identified in canine MDR isolates from different countries [1,11,14,15,28,29,30,33,34,37,38,39,40,41,42,66,68,71,72,73,75,76,77,80,81,84,85,86,87,88]. In contrast to some studies, we did not detect either canine MDR isolates displaying the five important emerging MDR STs in humans: ST69, ST127, ST131, ST410, and ST1193 [1,2,11,14,15,17,27,28,29,30,31,32,33,34,35,36,38,39,40,41,70,71,74,78], canine isolates harbouring the *mcr*-1 gene encoding resistance to colistin as described in China [89], or canine isolates producing carbapeneamases, notably OXA-48, as described in Germany [90], France [91], and United States [38]. Finally, we found that none of the 29 here studied ST372 was MDR while previous studies have found ST372 isolates producing different types of ESBLs and CMY-2 [1,11,14,30,33,38,40,41,42,79].

To get more insight into the potential link between the canine ExPEC and/or UPEC and the *E. coli* isolates causing extra-intestinal infections in humans, we determined which clone (defined by the association of phylogroup, clonotype and ST) and which serotypes characterized the 91 canine ExPEC and/or UPEC in order to compare them with human *E. coli* clinical isolates collected in 2015 and 2016 in Spain and France and characterized for these two traits [3,21,62]. This approach allowed us to found that among the 50 clones identified in the 91 Spanish canine ExPEC and/or UPEC isolates, 15 were present in the human collection accounting for 49 (18,8%) of the human isolates. However, only 31 of the 49 human ExPEC and/or UPEC isolates presented the same O:H serotype as the canine ones. By coupling clonal type and serotype for each *E. coli* ST lineage shared by dogs and humans, we observed various features about the distribution of the human isolates when the lineages included several clones and several clone-related serotype in dogs. This feature shows that it is difficult to make hypotheses about the relationship between canine and human isolates sharing a given clone-serotype couple in a given lineage without knowing the structure of the clone-serotype couples in the given lineage in humans. For example, we had found [3] that the human ST73 isolates were distributed into four clones, of which two here were identified in dogs. In human, three of the four clones comprised, each, isolates with different serotypes but one serotype (O6:H1) was exhibited by isolates distributed into the four clones. In dogs, the ST73 isolates exhibited only serotype O6:H1. This suggests that serotype might be an ecological niche marker, meaning, in this case, that isolates of the lineage ST73 exhibiting serotype O6:H1 are adapted to both dogs and humans. However, the Kidsley et al.’s study [10] in which a phylogenetic tree was built with the genome of ST73 strains from dogs, cats, and humans, seems to contradict this hypothesis. Indeed, the 13 studied Australian canine isolates of the lineage ST73 exhibited four serotypes among which serotype O6:H1 was exhibited by only one isolate that formed an animal-specific cluster (containing cat O6:H1 ST73 isolates) distinct from the four main clusters of human O6:H1 ST73 isolates. Nevertheless, the hypothesis that we made for serotype O6:H1 with regard to Spanish canine and human ST73 isolates could be made for serotype O2:H1 with regard to Australian canine and human ST73 isolates as this serotype was shared by clustered canine and human isolates. By extending the comparison of the structure of clone-serotype couples to the other human-specific-human ST lineages (ST127, ST141 and ST1193) shared by the Spanish studied dogs and humans, we observed that the clone-serotype couples shared by dogs and humans comprised mostly the serotype the most frequent in the human clones. This feature seems to indicate that serotype frequency might be a variable involved in the *E. coli* exchanges between dogs and humans. Concerning the dog-specific lineage ST372, we had found only one clone comprising to isolates in Spanish humans, while we found here five clones in the 29 Spanish dogs. Among the six serotypes exhibited by these 29 canine isolates, the serotype exhibited by the two human ST372 isolates corresponded to one (O18:H31) of the two dominant serotypes in dogs that was, on the other hand, exhibited by canine isolates belonging to three different clones. Thus, the suggestions that we made about the fact that serotype could be an ecological niche marker and that serotype frequency could shape the *E coli* exchange between dogs and humans seems to be able to be applied to the lineage ST 372.

Interestingly, concerning the 24 clones identified in the 28 canine MDR isolates, which were mostly non-B2-group isolates, we observed that if there were some clones (n = 9) shared by the Spanish human (35 of 394) and canine (10 of 197) isolates there was only one isolate that shared the same clone and the same serotype as one canine isolate.

To better understand the potential relationship between canine and human *E. coli* isolates with regard to the lineage ST372, we turned to the whole genome sequencing and analysis of 197 ST372 strains (151 from dogs and 46 from humans). The SNP analysis of the core genome of these 197 strains revealed an extensive phylogenetic diversity of the ST372 isolates that was segregated into six clusters. Cluster 1 comprised 91.4% of canine strains while cluster 2 comprised 60.9% of human strains. Cluster 2 was specific of human strains associated with serotypes O18:H31 and O45:H31, the latter serotype being exclusively found in human ST372 strains. Three other serotypes were the most prevalent serotype among strains belonging to cluster 1, including O4:H31 and O15:H31 associated with canine strains, and O83:H31 identified in similar proportion among canine and human strains. Overall, the WGS analysis suggests that canine strains of clone B2-CH103-9-ST372, belonging to cluster 1 and having serotype O83:H31 might cause extraintestinal infections in humans and dogs, as already suggested by the clone-serotype couple analysis, whereas strains of this clone belonging to cluster 2 and having serotypes O18:H31 and O45:H31 might cause only human extraintestinal infections. Molecular epidemiological studies on *E. coli* ST372 in human extra-intestinal infections are required to confirm these suggestions.

Furthermore, we localized ICEs in the chromosome of 22 of the ST372 sequenced genomes and confirmed that all ICEs belong to a yersiniabactin synthesis-associated ICE type (ICEKp1 family) with relaxase type MOB_Q._ In contrast, we found very few plasmids. Moreover, we found that the number of plasmids retrieved from the human ST372 strains was higher than that of plasmids found in the canine strains (four plasmids in the two human strains versus seven plasmids in the 21 canine strains). Interestingly, the genome of canine LREC_356 strain from cluster 4 carried two plasmids and was the canine strain genome the most similar to human strain genomes. Those plasmids were not rich with genes encoding of antibiotic resistance and virulence-factors. Nonetheless, a high number of virulence factor encoding-genes were found in the chromosome of the ST372 genomes and we hypothesized that the origin of the UPEC status of ST372 strains is due to the acquisition of ICEs harbouring the genes associated with this status. Although there is still limited knowledge about the origin of genomic islands, like ICEs or pathogenicity islands (PAIs), it has been speculated that they derive from the integration of plasmids or phages into the chromosome. Further, genomic research has shown that genomic islands have played a major role in the transformation of avirulent into virulent bacteria. Besides, most VFs of ExPEC are encoded by ICEs and PAIs [92,93,94,95].

## 5. Conclusions

The intestinal tract of healthy dogs appears as an important reservoir of ExPEC and/or UPEC, and, in a lesser extent, of MDR *E. coli* isolates. However, the canine MDR isolates could be a good reservoir of ESBLs and CMY-2 because most of them produce these enzymes. Among the canine isolates displaying an ExPEC and/or UPEC status, clone B2-CH103-9-ST372 was dominant. This canine clone and 14 others, also displaying an ExPEC and/or UPEC status, had been identified in isolates previously published as causing extraintestinal infections in human suggesting a zoonotic potential of these clones. WGS analysis suggests that canine strains of clone B2-CH103-9-ST372, belonging to cluster 1 and having serotype O83:H31 might cause extraintestinal infections in both humans and dogs, whereas those strains of this clone belonging to cluster 2 and serotypes O18:H31 and O45:H31 might cause only human infections. Taking into consideration that Kidsley et al. have recently characterized the phylogenetic relationship between canine, cat, and human isolates of the lineage ST73 [10], such studies are still required for the other ST lineages and clones that we showed in this study to be shared by canine and human isolates in order to clarify their potential role in infection occurrence in both dogs and humans.

## Figures and Tables

**Table 1:** Virulence factor (VF)-encoding genes detected in the 197 canine *E. coli* isolates. Relationship with phylogenetic groups.

VF-Encoding Gene	Number of Isolates (%)									
	Total (n = 197)	A (n = 32)	B1 (n = 26)	B2 (n = 84)	C (n = 10)	D (n = 6)	E (n = 14)	F (n = 18)	Clade V (n = 1)	NT ^1^ (n = 6)
Adhesins										
*fimH*	192 (97.5)	29	24	84	10	6	14	18	1	6
*fimAvMT78*	20 (10.2)	12	1	1	0	0	2	3	0	1
*papAH*	62 (31.5)	2	3	54	0	1	1	1	0	0
*papC*	62 (31.5)	2	3	54	0	1	1	1	0	0
*sfa/focDE*	66 (33.5)	0	2	63	0	0	0	1	0	0
*afa/draBC*	1 (0.5)	0	0	0	0	1	0	0	0	0
*yfcV*	96 (48.7)	4	0	80	0	0	0	11	0	1
Toxins										
*sat*	3 (1.5)	0	0	2	0	0	0	1	0	0
*cnf1*	54 (27.4)	0	0	53	0	0	0	1	0	0
*hlyA*	56 (28.4)	0	2	54	0	0	0	0	0	0
*cdtB*	8 (4.1)	0	0	6	0	0	0	2	0	0
*tsh*	21 (10.7)	2	2	0	8	0	2	6	0	1
*vat*	79 (40.1)	1	0	76	0	0	0	2	0	0
Iron uptake										
*iutA*	33 (16.8)	3	3	7	8	1	2	9	0	0
*iroN*	93 (47.2)	3	6	66	8	0	2	8	0	0
*fyuA*	106 (53.8)	7	2	77	8	5	1	5	0	1
*chuA*	122 (61.9)	0	0	84	0	6	14	18	0	0
Capsule										
*kpsM II*	64 (32.5)	1	0	41	0	4	4	10	1	3
*neuC-K1*	12 (6.1)	0	0	10	0	0	0	2	0	0
*kpsM II-K2*	7 (3.6)	0	0	2	0	3	0	1	0	1
*kpsM II-K5*	45 (22.8)	1	0	29	0	1	4	7	1	2
*kpsM III*	6 (3.0)	1	0	2	0	2	1	0	0	0
Miscellaneous										
*cvaC*	21 (10.7)	1	4	2	8	0	0	6	0	0
*iss*	31 (15.7)	2	5	6	8	0	2	8	0	0
*traT*	59 (29.9)	2	12	17	8	2	7	10	0	1
*ibeA*	54 (27.4)	0	0	49	0	0	0	3	0	2
*malX*	93 (47.2)	0	5	79	0	0	0	7	0	2
*usp*	86 (43.7)	1	3	77	0	1	1	2	0	1
ExPEC status	74 (37.6)	2	2	61	0	1	1	7	0	0
UPEC status	82 (41.6)	1	0	77	0	0	0	4	0	0
Range of VFs	0 to 18	0 to 10	1 to 10	2 to 17	1 to 8	5 to 7	2 to 7	3 to 18	3	1 to 9
Mean of VFs	7.87	2.31	2.96	12.79	6.60	5.67	4.14	7.94	3.00	3.67

^1^ Using the revised protocol developed by Clermont et al. [43] six isolates were not typeable (NT). These six isolates belonged to phylogroup A using the first protocol developed by Clermont et al. [43] that classifies isolates into only four phylogenetic groups (A, B1, B2, D).

**Table 2:** Virulence factor (VF)-encoding genes detected in the 65 canine *E. coli* isolates included in the 7 most frequent sequence types identified in ExPEC, UPEC and MDR isolates.

VF-Encoding Gene	Number of Isolates						
	B2-ST12 (n = 9)	D-ST38 (n = 4)	B2-ST73 (n = 4)	B2-ST127 (n = 8)	B2-ST141 (n = 5)	B2-ST372 (n = 29)	F-ST648 (n = 6)
Adhesins							
*fimH*	9	4	4	8	5	29	6
*fimAv_MT78_*	0	0	0	0	0	0	0
*papAH*	9	0	4	7	1	21	0
*papC*	9	0	4	7	1	21	0
*sfa/focDE*	9	0	3	8	4	26	1
*afa/draBC*	0	0	0	0	0	0	0
*yfcV*	9	0	4	8	5	29	6
Toxins							
*sat*	0	0	0	0	0	0	0
*cnf1*	8	0	4	7	0	23	0
*hlyA*	9	0	4	7	1	23	0
*cdtB*	0	0	2	0	0	0	1
*tsh*	0	0	0	0	0	0	5
*vat*	8	0	4	8	5	29	2
Iron uptake							
*iutA*	1	0	0	1	0	0	6
*iroN*	9	0	2	7	4	26	5
*fyuA*	8	4	4	8	5	29	3
*chuA*	9	4	4	8	5	29	6
Capsule							
*kpsM II*	6	4	4	7	5	2	6
*neuC-K1*	0	0	0	0	5	0	1
*kpsM II-K2*	0	3	0	0	0	0	1
*kpsM II-K5*	6	1	4	7	0	2	4
*kpsM III*	2	0	0	0	0	0	0
Miscellaneous							
*cvaC*	0	0	0	0	0	0	5
*iss*	0	0	0	1	0	0	5
*traT*	1	1	2	1	0	4	6
*ibeA*	0	0	0	0	4	29	1
*malX*	9	0	4	8	5	29	2
*usp*	9	1	4	7	5	29	1
ExPEC status	9	0	3	8	4	20	6
UPEC status	8	0	3	8	5	29	3
Range of VFs	12 to 16	5 to 7	14 to 16	12 to 17	10 to 14	8 to 16	9 to 18
Mean of VFs	14.40	5.50	15.30	14.40	12.00	13.10	12.17

**Table 3:** Clones and clonal-related serotypes of 91 canine ExPEC and/or UPEC isolates. Prevalence of the canine clones and clonal-related serotypes among ExPEC and/or UPEC isolates causing extraintestinal infections in humans [3,21,62].

Clone of Canine ExPEC and/or UPEC Isolates	Clone-Related Serotype of Canine ExPEC and/or UPEC Isolates (Number of Isolates)	Number of Human ExPEC and/or UPEC Isolates with Same Clone of Canine Isolates (49 of 261)	Number of Human ExPEC and/or UPEC Isolates with Same Clone and Serotype of Canine Isolates (31 of 261)
**A-CH11-NEG-ST93**	O5:H4 (1)	3	0
A-CH11-27-ST new 1	O4:H27 (1)	0	0
B1-CH4-27-ST58	O8:H25 (1), O9:H25 (1)	0	0
B2-CH13-5-ST12	O4:HNM (1), O18:H5 (1)	0	0
B2-CH13-7-ST12	O4:H1 (1), O4:HNM (1)	0	0
B2-CH13-130-ST12	O18:H5 (1)	0	0
**B2-CH13-223-ST12**	O18:H5 (2)	1	0
B2-CH13-430-ST12	O4:H5 (1)	0	0
B2-CH13-431-ST12	O4:H5 (1)	0	0
B2-CH24-9-ST73	O120:H31 (1)	0	0
**B2-CH24-27-ST73**	**O6:H1** (1)	1	1
**B2-CH24-30-ST73**	**O6:H1** (1)	4	3
**B2-CH24-103-ST73**	**O6:H1** (1)	6	4
B2-CH24-1-ST80	O75:H7 (1)	0	0
**B2-CH38-30-ST95**	O1:H7 (1)	1	0
**B2-CH14-2-ST127**	**O6:HNM** (2)	4	3
**B2-CH14-180-ST127**	**O6:HNM** (3), O6:H11 (1)	1	1
B2-CH14-fimH_TR_new 1-ST127	O6:H31 (1), O6:HNM (1)	0	0
B2-CH40-NEG-ST131	O25:H4 (1)	0	0
**B2-CH52-5-ST141**	**O2:H6** (1)	13	9
**B2-CH52-14-ST141**	**O2:H6** (4)	2	2
**B2-CH103-9-ST372**	O4:H31 (9), O15:H31 (1), O21:H31 (3), O25:H31 (4), **O83:H31** (7), O117:H28 (1)	2	2
B2-CH103-10-ST372	O15:H31 (1)	0	0
B2-CH103-17-ST372	O15:H31 (1)	0	0
B2-CH103-240-ST372	O83:H31 (1)	0	0
B2-CH103-706-ST372	O83:H31 (1)	0	0
B2-CH96-433-ST646	O22:HNM (1)	0	0
B2-CH43-fimH_TR_new 2-ST929	O138:H14 (1)	0	0
B2-CH13-175-ST961	O4:HNM (1)	0	0
B2-CH52-428-ST998	O2:H6 (1)	0	0
**B2-CH14-64-ST1193**	**O75:HNM** (2)	4	3
B2-CH363-75-ST2622	O83:H6 (1)	0	0
B2-CH195-2-ST5644	O175:H5 (1)	0	0
B2-CH13-fimH_TR_new 3-ST new 2	O18:HNM (1)	0	0
B2-CH13-429-ST new 3	O4:H5 (1)	0	0
B2-CH103-9-ST new 4	O4:H31 (1)	0	0
B2-CH103-12-ST new 5	O6:HNM (1)	0	0
B2-CH11-34-ST new 6	O5:H11 (1)	0	0
B2-CH40-20-ST new 7	O1:H4 (1)	0	0
B2-CH363-75-ST new 8	O83:H4 (1)	0	0
B2-CH24-2-ST new 9	ONT:H1 (1)	0	0
B2-CH24-1473-ST new 10	O120:H5 (1)	0	0
B2-CH23-31-ST new 11	O103:H4 (1)	0	0
B2-CH40-20-ST new 12	O1:H4 (1)	0	0
B2-CH40-21-ST new 13	O13:H4 (1)	0	0
D-CH35-27-ST new 14	O77:H18 (1)	0	0
E-CH132-65-ST501	ONT:H1 (1)	0	0
**F-CH32-41-ST59**	**O1:H7** (1)	4	3
**F-CH4-27-ST648**	O4:H6 (1)	1	0
**F-CH4-58-ST648**	O153:H42 (5)	2	0

Bold highlights those canine clones and clone-related serotypes also detected among ExPEC and/or UPEC isolates causing extraintestinal infections in humans.

**Table 4:** Clones and clonal-related serotypes of 28 canine multidrug resistant (MDR) *E. coli* isolates. Prevalence of the canine clones and clone-related serotypes among *E. coli* isolates causing extraintestinal infections in humans [3,21,62].

Clone of Canine MDR Isolates	Clone-Related Serotype of Canine MDR Isolates (Number of Isolates)	Type of ESBL and pAmpC Enzymes Produced by Canine MDR Isolates	Number of Human *E. coli* Isolates with Same Clone of Canine MDR Isolates (Number and Type ESBL Produced by Human Isolates) (35 of 394)	Number of Human *E. coli* Isolates with Same Clone and Serotype of Canine MDR Isolates (1 of 394)
**A-CH11-54-ST10**	O128:HNM (1)	SHV12	10 (3 SHV12)	0
**A-CH11-NEG-ST93**	O5:H4 (1)	none	4 (1 CTX-M-14)	0
A-CH11-54-ST8953	O101:HNM (1)	CMY-2	0	0
A-CH11-27-ST new 1	O4:H27 (1)	none	0	0
**B1-CH4-27-ST58**	O8:H25 (1), **O9:H25** (1)	none	4 (1 CTX-M-14 and 1 CTX-M-32)	1
B1-CH4-121-ST155	O5:H11 (1)	none	0	0
**B1-CH4-366-ST155**	O9:H10 (1)	CMY-2	1 (1 CTX-M-1)	0
B1-CH4-425-ST new 15	O123:H11 (1)	CTX-M-1	0	0
B1-CH4-31-ST new 16	O8:H7 (1)	CTX-M-1	0	0
B1-CH29-38-ST new 17	O8:H49 (1)	CTX-M-1	0	0
B1-CH30-38-ST new 18	O12:H8 (1)	CMY-2	0	0
**B2-CH13-223-ST12**	O18:H5 (1)	CMY-2	1	0
B2-CH13-429-ST new 3	O4:H5 (1)	CMY-2	0	0
**C-CH4-39-ST88**	O45:HNM (1)	CTX-M-1	11 (1 CTX-M-14)	0
D-CH26-5-ST38	O86:H18 (3)	CTX-M-14	0	0
**D-CH26-65-ST38**	O1:H34 (1)	CMY-2	2 (1 CTX-M-15)	0
D-CH35-27-ST new 14	O77:H18 (1)	none	0	0
**E-CH31-54-ST57**	O27:H40 (1)	CMY-2	1	0
E-CH11-167-ST695	O99:H38 (1)	none	0	0
E-CH4-31-ST1011	O166:H45 (1)	CTX-M-55	0	0
E-CH23-221-ST1140	O44:H39 (1)	none	0	0
E-CH485-426-ST3774	O9:H31 (1)	CMY-2	0	0
F-CH88-145-ST457	O11:H25 (2)	CMY-2	0	0
**F-CH4-27-ST648**	O4:H6 (1)	CTX-M-14	1 (1 CTX-M-15)	0

Bold highlights those canine clones and serotypes also detected among *E. coli* isolates causing extraintestinal infections in humans.

**Table 5:** Distribution into the phylogenetic clusters of the 197 canine and human ST372 strains.

Cluster	Number of Strains (%)		*p*-Value ^1^
	Canine (n = 151)	Human (n = 46)	
1	138 (91.4)	9 (19.6)	<0.00001
2	0	28 (60.9)	<0.00001
3	0	3 (6.5)	0.01209
4	9 (6.0)	0	
5	0	3 (6.5)	0.01209
6	2 (1.3)	1 (2.2)	
Undefined	2 (1.3)	2 (4.3)	

^1^ Two-tailed *p* values by Fisher’s exact probability test are shown where *p* < 0.05.

**Table 6:** Cluster distribution of the 197 studied ST372 strains according to countries.

Cluster (Number of Strains)	Number of Strains (%)		*p*-Value ^1^ Europe vs. North America	Countries (Number of Strains)
	Europe (n = 46)	North America (n = 143)		
1 (n = 147)	30 (65.2)	117 (81.8)	0.02476	USA (109), Spain (20), Canada (6), UK (4), Sweden (3), France (2), Germany (1), North America (2)
2 (n = 28)	11 (23.9)	13 (9.1)	0.01233	USA (13), UK (8), Spain (1), France (1), The Netherlands (1), Australia (2), unknown (2)
3 (n = 3)	0	0		Japan (1), Kenya (1), unknown (1)
4 (n = 8)	1 (2.2)	7 (4.9)		USA (7), Spain (1), unknown (1)
5 (n = 3)	3 (6.5)	0	0.01371	Denmark (2), France (1)
6 (n = 3)	1 (2.2)	2 (1.4)		USA (2), UK (1)
Undefined (n = 5)	0	4 (2.8)		USA (4), Australia (1)

^1^ Two-tailed *p* values by Fisher’s exact probability test are shown where *p* < 0.05.

**Table 7:** Distribution of the VF-encoding genes detected among the 197 ST372 *E. coli* genomes according to strain origins (canine/human) and cluster types.

VF-Encoding Gene	Number of Strains (%)									*p*-Value ^1^ Canine vs. Human
	Canine (n =151)	Human (n = 46)	Cluster 1 (n = 147)	Cluster 2 (n = 28)	Cluster 3 (n = 3)	Cluster 4 (n = 9)	Cluster 5 (n = 3)	Cluster 6 (n = 3)	Undefined (n = 4)	
Adhesins										
*fimH*	151 (100)	46 (100)	147	28	3	9	3	3	4	
*papAH*	88 (58)	5 (11)	89	0	0	0	0	3	1	<0.00001
*papC*	113 (75)	9 (20)	113	0	0	0	3	3	3	<0.00001
*papEF*	112 (74)	9 (20)	113	0	0	0	3	2	3	<0.00001
*sfaDE*	3 (2)	0 (0)	1	0	0	0	0	2	0	
*sfaS*	6 (4)	0 (0)	6	0	0	0	0	0	0	
*focCD*	136 (90)	12 (26)	144	0	0	0	1	1	2	<0.00001
*focG*	138 (91)	12 (26)	144	0	0	0	1	3	2	<0.00001
*afaBCD/draP*	0	0 (0)	0	0	0	0	0	0	0	
*yfcV*	150 (99)	46 (100)	146	28	3	9	3	3	4	
Toxins										
*sat*	0	0 (0)	0	0	0	0	0	0	0	
*cnf1*	117 (77)	11 (24)	120	0	0	0	3	3	2	<0.00001
*hlyA*	117 (77)	11 (24)	119	0	0	0	3	3	3	<0.00001
*hlyF*	1 (1)	3 (7)	1	0	0	0	3	0	0	0.04034
*cdtB*	0	0 (0)	0	0	0	0	0	0	0	
*vat*	148 (98)	45 (98)	144	27	3	9	3	3	4	
Iron uptake										
*iutA*	1 (1)	3 (7)	1	0	0	0	3	0	0	0.04034
*iroN*	139 (92)	14 (30)	144	0	0	0	3	3	3	<0.00001
*fyuA*	151 (100)	46 (100)	147	28	3	9	3	3	4	
*chuA*	151 (100)	46 (100)	147	28	3	9	3	3	4	
*ireA*	5 (3)	1 (2)	5	0	0	0	0	0	1	
Capsule										
*kpsM II*	16 (11)	36 (78)	5	28	3	9	3	0	4	<0.00001
*kpsM II-K1*	0 (0)	0 (0)	0	0	0	0	0	0	0	
*kpsM II-K2*	0 (0)	0 (0)	0	0	0	0	0	0	0	
*kpsM II-K5*	16 (11)	36 (78)	5	28	3	9	3	0	4	<0.00001
Miscellaneous										
*iss1*	1 (1)	3 (7)	1	0	0	0	3	0	0	0.04034
*iss2*	140 (93)	45 (98)	136	27	3	9	3	3	4	
*traT*	34 (23)	10 (22)	25	5	0	9	3	1	1	
*ibeA*	149 (99)	46 (100)	146	28	3	9	3	3	3	
*malX-PAI*	150 (99)	46 (100)	146	28	3	9	3	3	4	
*usp*	151 (100)	46 (100)	147	28	3	9	3	3	4	
*ompT*	151 (100)	46 (100)	147	28	3	9	3	3	4	
ExPEC status	113 (75)	10 (22)	113	0	1	0	3	3	3	<0.00001
UPEC status	151 (100)	46 (100)	147	28	3	9	3	3	4	
Mean of VFs	16.79	13.76	16.93	12.11	12.00	13.00	21.67	18.00	17.00	

^1^ Two-tailed *p* values by Fisher’s exact probability test are shown where *p* < 0.05.

**Table 8:** Distribution of serotypes among the 197 ST372 strains according to origins (canine and human) and cluster types.

Serotype In Silico	Number of Strains (%)		*p*-Value ^1^ Canine vs. Human	Number of Isolates Belonging to Cluster						
	Canine (n = 151)	Human (n = 46)								
				1	2	3	4	5	6	Undefined
O2:H6	1 (0.7)	0								1
O4:H31	32 (21.2)	3 (6.5)	0.02631	35						
O6:H31	2 (1.3)	1 (2.2)							3	
O15:H31	30 (19.9)	0	0.00062	30						
O18:H31	0	20 (43.5)	<0.00001		20					
O21:H14	5 (3.3)	0		5						
O21:H31	4 (2.6)	1 (2.2)		5						
O25:H31	4 (2.6)	0		4						
O45:H31	0	6 (13.0)	0.00012		6					
O75:H31	5 (3.3)	0		5						
O83:H31	58 (38.4)	13 (28.3)		53		3	9	3		3
O117:H28	6 (4.0)	0		6						
O-unknown:H31	3 (2.0)	2 (4.3)		3	2					
O-unknown:H28	1 (0.7)	0		1						

^1^ Two-tailed *p* values by Fisher’s exact probability test are shown where *p* < 0.05.

**Table 9:** Serotypes of the 197 ST372 strains according to countries.

Serotype in Silico	Number of Strains (%)		*p*-Value ^1^ Europe vs. North America	Countries (Number of Strains)
	Europe (n = 46)	North America (n = 143)		
O2:H6	0	1 (0.7)		USA (1)
O4:H31	11 (23.9)	24 (16.8)		USA (23), Spain (7), UK (2), France (1), Sweden (1), Canada (1)
O6:H31	1 (2.2)	2 (1.4)		USA (2), UK (1)
O15:H31	4 (8.7)	26 (18.2)		USA (25), Spain (3), Canada (1), France (1)
O18:H31	9 (19.6)	10 (7.0)	0.02203	USA (10), UK (7), Spain (1), France (1)
O21:H14	1 (2.2)	4 (2.8)		USA (4), Sweden (1)
O21:H31	3 (6.5)	2 (1.4)		Spain (2), UK (1), USA (1), Canada (1)
O25:H31	4 (8.7)	0	0.00317	Spain (4)
O45:H31	1 (2.2)	2 (1.4)		USA (2), Australia (2), Netherlands (1)
O75:H31	0	5 (3.5)		USA (5)
O83:H31	10 (21.7)	57 (39.9)	0.03291	USA (52), Spain (4), Canada (2), Denmark (2), UK (1), France (1), Sweden (1), Germany (1)
O117:H28	1 (2.2)	5 (3.5)		USA (5), Spain (1)
O-unknown:H31	1 (2.2)	4 (2.8)		USA (3), Canada (1), UK (1)
O-unknown:H28	0	1 (0.7)		USA (1)

^1^ Two-tailed *p* values by Fisher’s exact probability test are shown where *p* < 0.05.

**Table 10:** Description of mobile genetic elements (MGE)s found in the 23 ST372 strain genomes sequenced in this study.

Genome of Strain	Serotype of Strain	Origin	Cluster	MGEs		MOB/Inc Typing; Size (kb)	
				ICEs (kb of Contig)	Number of Plasmids	Plasmid_1	Plasmid_2
LREC_341	O18:H31	Human	2	MOB_Q_ (308)	2	MOB_P1_/nd ^1^; (164)	nd/p0111; (92)
LREC_342	O18:H31	Human	2	MOB_Q_ (90)	2	MOB_P1_/nd; (720)	MOB_Qu_/ColRNAI; (4)
LREC_343	O4:H31	Canine	1	MOB_Q_ (474)	1	MOB_F12_/IncFII [F-:A-:B-], pCD1; (66)	
LREC_344	O4:H31	Canine	1	MOB_Q_ (1208)	0		
LREC_345	O4:H31	Canine	1	MOB_Q_ (158)	1	MOB_F12_/IncFII [F2:A-:B-], pCD1; (75)	
LREC_346	O4:H31	Canine	1	MOB_Q_ (653)	1	MOB_P3_/IncX1; (47)	
LREC_347	O117:H28	Canine	1		1	MOB_P3_/IncX1; (38)	
LREC_348	O4:H31	Canine	1	MOB_Q_ (472)	0		
LREC_349	O83:H31	Canine	1	MOB_Q_ (707)	0		
LREC_350	O83:H31	Canine	1	MOB_Q_ (707)	0		
LREC_351	O4:H31	Canine	1	MOB_Q_ (653)	0		
LREC_352	O25:H31	Canine	1	MOB_Q_ (658)	0		
LREC_353	O25:H31	Canine	1	MOB_Q_ (658)	0		
LREC_354	O25:H31	Canine	1	MOB_Q_ (658)	0		
LREC_355	O4:H31	Canine	1	MOB_Q_ (157)	0		
LREC_356	O83:H31	Canine	4	MOB_Q_ (2541)	2	MOB_F12_/IncFIB, IncFII [F-:A-:B52]; (162)	MOB_P3_/IncX1; (36)
LREC_357	O83:H31	Canine	1	MOB_Q_ (1678)	0		
LREC_358	O25:H31	Canine	1	MOB_Q_ (660)	0		
LREC_359	O21:H31	Canine	1	MOB_Q_ (1317)	0		
LREC_360	O15:H31	Canine	1	MOB_Q_ (86)	0		
LREC_361	O21:H31	Canine	1	MOB_Q_ (1317)	0		
LREC_362	O15:H31	Canine	1	MOB_Q_ (641)	0		
LREC_363	O15:H31	Canine	1	MOB_Q_ (172)	1	MOB_H11_/IncHI2; (204)	

^1^ nd, not detected.

## Supplementary Materials

The following are available online at https://www.mdpi.com/2076-2607/8/11/1712/s1, Figure S1: Comparison of contigs harbouring integrative conjugative elements (ICEs) from 22 ST372 *E. coli* genomes, Table S1: Bioproject accession (PRJNA627579) and assembly genome information, Table S2: SNP matrix and VF-encoding genes of 197 ST372 genomes, Table S3: Prevalence of the phylogenetic groups in the 197 canine *E. coli* isolates, Table S4: Comparison of the distribution of the phylogenetic groups among the 197 canine isolates according to the strain ExPEC and UPEC status, Table S5: Comparison of the distribution of the phylogenetic groups among the 197 canine isolates according to the strain multidrug resistant (MDR) status, Table S6: Comparison of the strain ExPEC and UPEC status among canine multidrug resistant (MDR) and non-MDR isolates, Table S7: New sequence types observed in 18 canine *E. coli* isolates, Table S8: In silico determination of VF-encoding genes, antibiotic-resistance encoding genes (ARGs) and point mutations in 23 ST372 *E. coli* genomes.
