# Supplementary material for: Molecular Characteristics of Extraintestinal Pathogenic E. coli (ExPEC), Uropathogenic E. coli (UPEC), and Multidrug Resistant E. coli Isolated from Healthy Dogs in Spain. Whole Genome Sequencing of Canine ST372 Isolates and Comparison with Human Isolates Causing Extraintestinal Infections
Source: Microorganisms. 2020 Oct 31;8(11):1712. doi: 10.3390/microorganisms8111712 (PMC7716232; doi:10.3390/microorganisms8111712)
Supplement: Supplementary file 1 [file microorganisms-08-01712-s001.zip › microorganisms-946416-supplementary/microorganisms-946416-supplementary/Tables S3-S4-S5-S6-S7 and Figure S1.pdf]

Supplementary material

Molecular characteristics of extraintestinal pathogenic *E. coli* (ExPEC), uropathogenic *E. coli* (UPEC), and multidrug resistant *E. coli* isolated from healthy dogs in Spain. Whole genome sequencing of canine ST372 isolates and comparison with human isolates causing extraintestinal infections.

Saskia-Camille Flament-Simon [1,2], María de Toro[3], Vanesa García[1,2], Jesús E. *Bla*nco [1,2], Miguel *Bla*nco[1,2], María Pilar Alonso[2,4], Ana Goicoa[5,6], Juan Díaz-González[1], Marie-Hélène Nicolas-Chanoine[7], and Jorge *Bla*nco[1,2]*

[1] Laboratorio de Referencia de *E. coli* (LREC), Departamento de Microbioloxía e Parasitoloxía, Facultade de Veterinaria, Universidade de Santiago de Compostela (USC), Lugo, Spain.
[2] Instituto de Investigación Sanitaria de Santiago de Compostela (IDIS), Spain.
[3] Plataforma de Genómica y Bioinformática, Centro de Investigación Biomédica de La Rioja (CIBIR), Logroño, Spain.
[4] Unidade de Microbioloxía, Hospital Universitario Lucus Augusti (HULA), Lugo, Spain.
[5] Servicio de Medicina Interna, Hospital Veterinario Universitario Rof Codina, USC, Lugo, Spain.
[6] Departamento de Anatomía, Producción Animal e Ciencias Clínicas Veterinarias, USC, Lugo, Spain
[7] Université de Paris, IAME, UMR1137, INSERM, Paris, France.

Table S3. Prevalence of the phylogenetic groups in the 197 canine *E. coli* isolates.

**Phylogenetic group**	**Number of isolates (%)**
A	32 (16.2)
B1	26 (13.2)
B2	84 (42.6)
C	10 (5.1)
D	6 (3.0)
E	14 (7.1)
F	18 (9.1)
Clade V	1 (0.5)
Not typeable	6 (3.0)

Table S4. Comparison of the distribution of the phylogenetic groups among the 197 canine isolates according to the strain ExPEC and UPEC status.

**Phylogenetic group**	**Number of isolates (%)**		**P-value[1]**
	**ExPEC and/or UPEC (n= 91)**	**non-ExPEC and non-UPEC (n=106)**	
A	2 (2.2)	30 (28.3)	<0.00001
B1	2 (2.2)	24 (22.6)	0.00001
B2	78 (85.7)	6 (5.7)	<0.00001
C	0	10 (9.4)	0.00199
D	1 (1.1)	5 (4.7)	
E	1 (1.1)	13 (12.3)	0.00371
F	7 (7.7)	11 (10.4)	
Clade V	0	1 (0.9)	
Not typeable	0	6 (5.7)	0.03155

[1]Two-tailed *P* values by Fisher´s exact probability test are shown where $P < 0.05$.

Table S5. Comparison of the distribution of the phylogenetic groups among the 197 canine isolates according to the strain multidrug resistant (MDR) status.

**Phylogenetic group**	**Number of isolates (%)**		**P-value[1]**
	**MDR (n= 28)**	**non-MDR (n=169)**	
A	4 (14.3)	28 (16.6)	
B1	8 (28.6)	18 (10.7)	0.01597
B2	2 (7.1)	82 (48.5)	0.00004
C	1 (3.6)	9 (5.3)	
D	5 (17.9)	1 (0.6)	0.00022
E	5 (17.9)	9 (5.3)	0.03238
F	3 (10.7)	15 (8.9)	
Clade V	0	1 (0.6)	
Not typeable	0	6 (3.6)	

[1]Two-tailed *P* values by Fisher´s exact probability test are shown where $P < 0.05$.

Table S6. Comparison of the strain ExPEC and UPEC status among canine multidrug resistant (MDR) and non-MDR isolates.

**Status**	**Number of isolates (%)**		**P-value[1]**
	**MDR (n= 28)**	**non-MDR (n=169)**	
ExPEC	8 (28.6)	66 (39.1)	
UPEC	4 (14.3)	78 (46.2)	0.00156
ExPEC and/or UPEC	8 (28.6)	83 (49.1)	
Non ExPEC and non-UPEC	20 (71.4%)	86 (50.9%)	

[1]Two-tailed *P* values by Fisher´s exact probability test are shown where $P < 0.05$.

Table S7. New sequence types observed in 18 canine *E. coli* isolates.

**ST New**	**Allele numbers**						
	adk	***fum*C**	***gyr*B**	***icd***	***mdh***	***pur*A**	**recA**
1	10	11	4	666	560	8	2
2	13	13	9	13	16	[2]126-like	9
3	13	13	9	13	439	10	9
4	88	103	19	18	23	44	26
5	88	103	[1]19-like	36	23	44	26
6	6	11	4	8	235	8	2
7	13	40	9	13	16	126	9
8	13	363	302	97	23	94	93
9	13	24	9	912	17	11	25
10	36	24	10	912	17	10	25
11	9	23	33	628	11	8	6
12	13	40	459	912	23	28	109
13	13	40	13	912	23	25	66
14	21	35	27	6	286	5	4
15	6	4	4	16	474	8	14
16	6	4	5	18	11	319	6
17	6	29	33	402	11	7	2
18	63	30	3	628	43	350	6

[1]Nucleotide sequence of *gyrB* 19-like, closest match: *Escherichia coli* Achtman MLST *gyrB*: 19 (1 difference found: ^{49}C→^{49}T)

```
GGTCTGCACGGCGTTGGTGTTTCGGTAGTAAACGCCCTGTCGCAAAAATTGGAGCTGGTTATCCAGCGCGAGGGTAAAATTCACCGTCAGATCTACGAACA
CGGTGTACCGCAGGCCCCGCTGGCGGTTACCGGCGAGACTGAAAAAACCGGCACTATGGTGCGTTTCTGGCCAAGCCTTGAAACCTTCACCAATGTGACCG
AGTTCGAATATGACATTCTGGCGAAACGTCTGCGTGAGTTGTCGTTCCTCAACTCCGGCGTTTCCATTCGTCTGCGCGACAAGCGCGACGGCAAAGAAGAC
CACTTCCACTATGAAGGCGGCATCAAGGCGTTCGTTGAATATCTGAACAAGAACAAAACGCCGATCCACCCGAATATCTTCTACTTCTCCACCGAAAAAGA
CGGTATTGGCGTCGAAGTGGCGTTGCAGTGGAACGATGGCTTCCAGGAAAACATCT
```

[2]Nucleotide sequence of *purA* 126-like, closest match: *Escherichia coli* Achtman MLST *purA:* 126 (1 difference found: ^{118}A →^{118}G)

```
ATAACGCGCGTGAGAAAGCGCGTGGCGCGAAAGCGATCGGCACCACCGGTCGAGGTATCGGGCCTGCTTATGAAGATAAAGTGGCACGTCGCGGTCTGCGT
GTTGGCGACCTTTTCGGCAAAGAAACCTTCGCTGAAAAACTGAAAGAAGTGATGGAATATCACAACTTCCAGTTGGTTAACTACTACAAAGCTGAAGCGGT
TGATTACCAGAAAGTTCTGGATGATACGATGGCTGTTGCCGACATCCTGACTTCTATGGTTGTTGACGTTTCTGATCTGCTCGACCAAGCGCGTCAGCGTG
GCGATTTCGTCATGTTTGAAGGTGCGCAGGGTACGCTGCTGGATATCGACCACGGCACTTATCCGTACGTAACTTCTTCCAACACTACTGCTGGTGGCGTG
GCGACCGGTTCCGGCCTGGGCCCGCGTTATGTTGATTACGTTTTGGGTATCCTCAAAGCTTACTCCACTCGTGT
```

Figure S1. Comparison of contigs harbouring integrative conjugative elements (ICEs) from 22 ST372 *E. coli* genomes.

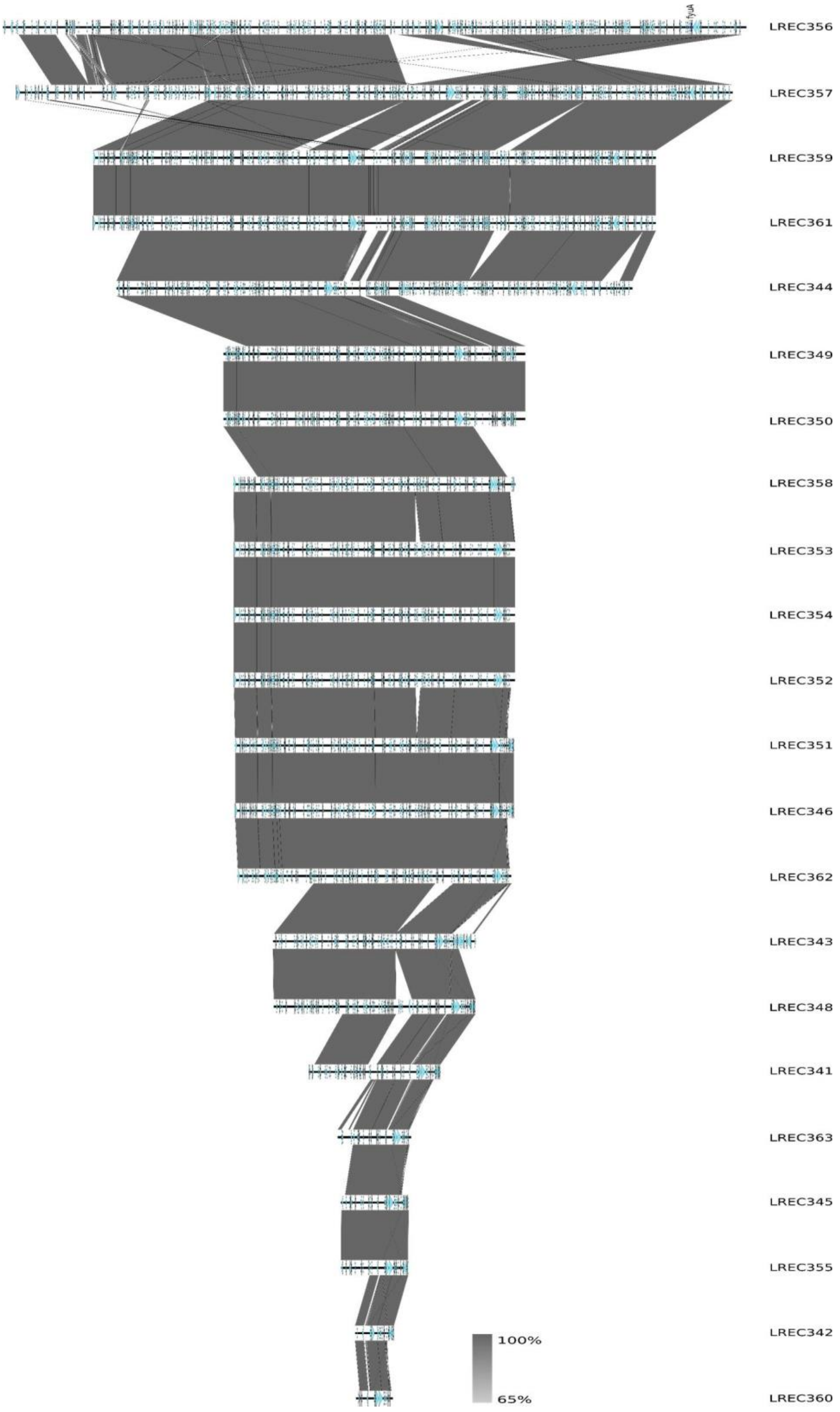

[1] The figure includes 22 complete contigs with a length ranging from 2,540,863 to 86,362 (pb). The shadow parallelograms denote genetic regions that exhibit sequence homology among different segments. Light shadow denotes regions with a lower level of sequence identity (99%) by BLAST. The image was generated using EasyFig [59] (default parameters).
